# Demonstration of Sarcocystis-like Parasites Found in Peripheral Blood

**Published:** 2014

**Authors:** Masomeh Bayani, Narges Kalantari, Majid Sharbatdaran, Zeinab Abedian, Salman Ghaffari

**Affiliations:** 1 Infectious Diseases and Tropical Medicine Research Center, Babol University of Medical Sciences, Babol, Iran.; 2 Cellular and Molecular Biology Research Center (CMBRC), Babol University of Medical Sciences, Babol, Iran.; 3 Laboratory Sciences Department, Faculty of Paramedical Sciences, Babol University of Medical Sciences, Babol, Iran.; 4 Pathology Department, Faculty of Medicine, Babol University of Medical Sciences, Babol, Iran.; 5 Parasitology and Mycology Department, Faculty of Medicine, Babol University of Medical Sciences, Babol, Iran.

**Fig. 1:**
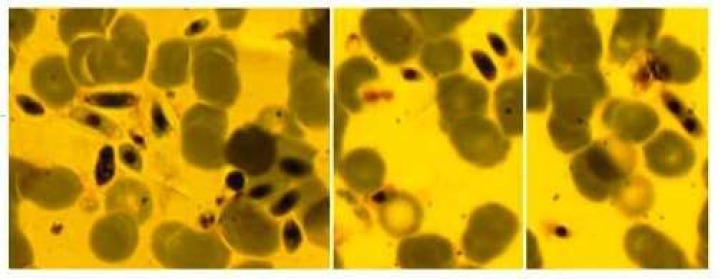
Merozoites of *Sarcocystis *sp on the blood smear of the patient (Giemsa stain, magnification x 1000).

Sir,

The tissue cyst-forming coccidian parasites, *Toxoplasma gondii *and *Sarcocystis *species are important in humans and animals. The genus *Sarcocystis* causes intestinal and muscular sarcocystosis infections in human. In the case of intestinal sarcocystosis, human served as a definitive host and infection is often asymptomatic and cleared spontaneously. Occasionally, nausea, loss of appetite, vomiting, stomach ache, bloat, diarrhea, dyspnea, and tachycardia may occur. In the case of muscular sarcocystosis, human becomes a dead-end intermediate host of *Sarcocystis *spp. of some carnivorous animals that prey on non-human primates (1). Recently, the International Society of Travel Medicine and the Center of Disease Control (CDC) have reported 32 suspected cases of acute muscular sarcocystosis among cases who recently traveled to malasia. The main symptoms were high fever and severe muscle pain, which started within days or weeks of returning home and had been prolonged. All subjects had peripheral eosinophilia and mostly had higher serum creatinine phosphokinase levels (2). More recently, Abubakar et al., have reported an outbreak of human infection with S.* nesbitti* in Malaysia and Makhaji reported two cases of muscular sarcocystosis in India (3-4). Nearly all human muscular sarcocystosis cases were recognized by the presence of intramuscular cysts. However, intravascular asexual stages have rarely been demonstrated in human muscular sarcocystosis patients (1, 5). There was only one report which demonstrated merozoites of* Sarcocystis *spp. in thin blood smear of a patient with acquired immunodeficiency syndrome (AIDS) who harbored intestinal forms of the parasite at the same time (6). In this report, we demonstrate an organism morphologically similar to merozoites of *Sarcocystis* spp. in the peripheral blood smear of an immunocompetent woman. The organisms were measured approximately 5 to 11 μm by 2 to 4 μm. They had blue cytoplasm with one or two nuclei and had different shapes. The parasitemia was 28 per 1000 red blood cells (Figure 1).

The woman was 47 years old and lived in a rural area. She was admitted to Rohani Hospital, Babol, northern Iran. Her symptoms were mild fever with a relapsing- remitting nature, rigor fatigue and muscle weakness in her arms, legs and paraspinal muscles. Her initial physical examination upon admission revealed a temperature of 38 °C; blood pressure, 120/80 mm Hg; respiratory rate, 20 breaths per minute and pulse, 80 beats per minute. The number of red blood cells, platelets and white blood cells were 5.05x10^6^ µl, 248000/µl and 5800/µl (54% neutrophil, 44% lymphocytes and 2% eosinophil), respectively. The hemoglobin and hematocrit levels were low and she had hypochrome anemia. The numbers of CD4 and CD8 lymphocytes were 1.535 and 1.017, respectively (CD4/ CD8 ratio: 1.51). 

The level of her serum immunoglobulin was normal and the amount of IgG, IgM and IgA was 13.3, 0.99 and 2.1 g/l, respectively. Lymphadeno-pathy was not seen and her serum was negative for anti- *T. gondii* antibodies (IgG and IgM) [ELFA technique using Mini-VIDAS kit (Biomerio, France)]. In order to confirm that the parasite belonged to *Sarcocystis* genus, serological tests including direct agglu-tination test (DAT) and indirect immunofluorescent test (IFAT) were performed. The DAT was carried out by *Sarcocystis* *moulei*. Zoites were obtained from macro-cysts in the skeletal muscles of goats as soon as the animals were slaughtered as previously described (7). 

Positive controls sera were obtained from mice sera (NMRI strain) which were experi-mentally inoculated intraperitoneally with a filtrate calf muscle sample containing micro-cysts of *Sarcocystis cruzi* (confirmed by PCR and sequence analyzing, Accession No. KC508514) (8). The IFAT was done as usual. Negative controls were un-inoculated mice sera and PBS. 

As the diagnosis of muscular *Sarcocystis *infection should be confirmed through microscopic examination of the muscle biopsy (9) and highly specific PCR, DNA extraction from the stained peripheral blood smear and PCR analysis of partial 18S rRNA using methods previously described, were performed (10-11). 

**Fig. 2:**
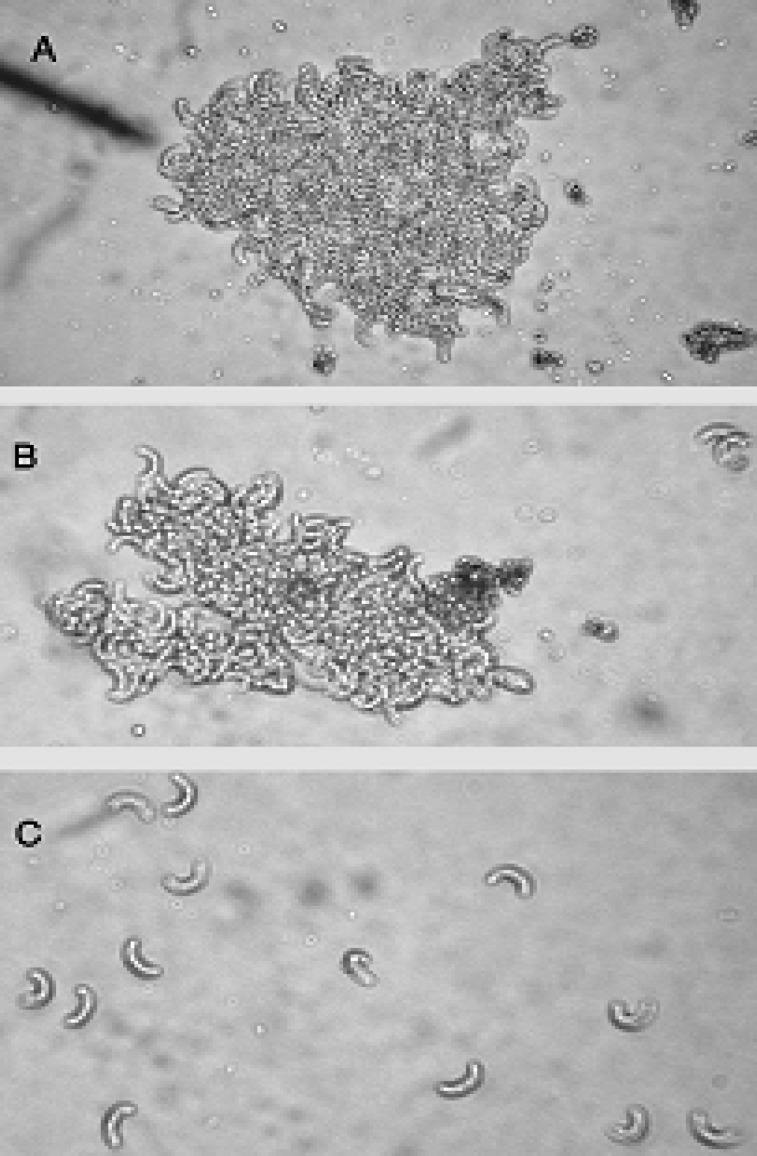
Photomicrographs of agglutinated or nonagglutinated zoites derived from goat muscles contaminated with macro-cysts of *S. moulei*. A) with a mice serum which was previously exposed to micro-cysts of *S. cruzi* as a positive control (1:20 dilution); B) with the patient serum (1:20 dilution); C) with negative sera and PBS. The test was read with the aid of light microscope and photos were taken at x 400 magnifications.

Findings obtained here showed that the parasites were agglutinated by the patient serum and the positive control. The parasites were not agglutinated by any negative control serum or PBS. The DAT was positive with titer of 1: 640 and 1: 1280 for the patient's serum and the two mice sera, respectively (Figure 2).

**Fig. 3:**
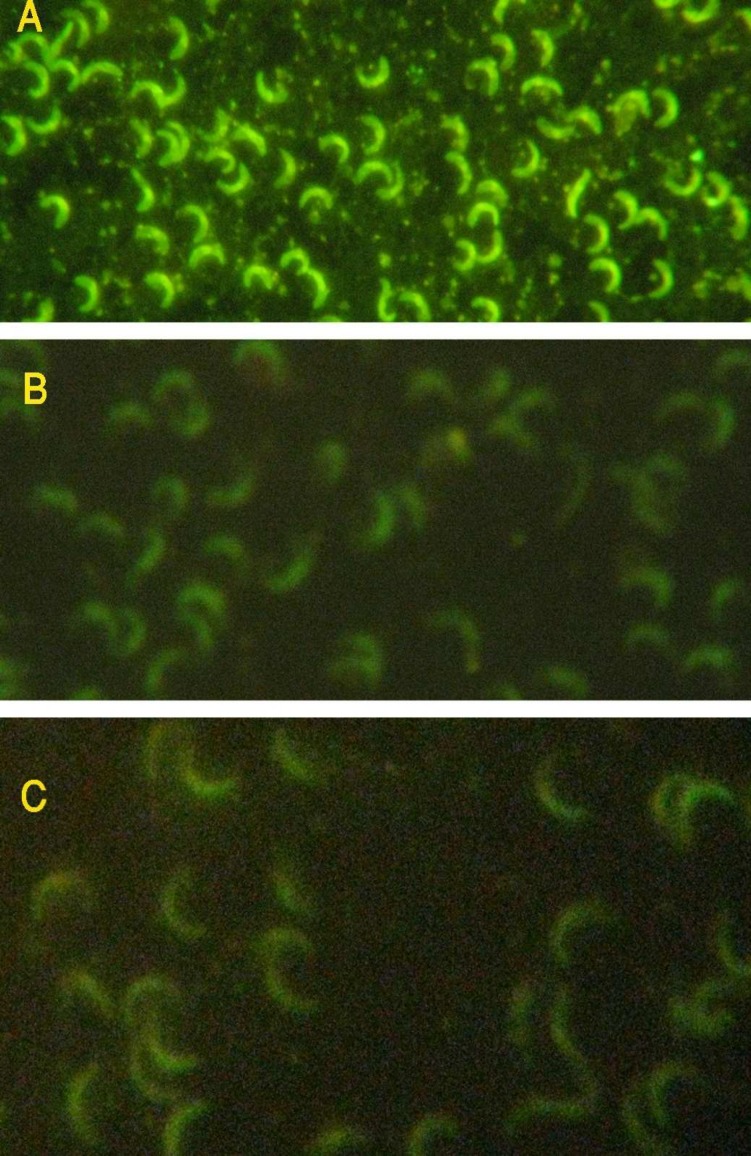
IFAT assay using zoites of *S. molei* derived from goat muscles. A) 1:160 dilution of the patient's serum; B) negative control; C) mounted antigen without treatment with any serum or PBS (autofluorescence). Note the strong reaction observed with the patient serum in comparison with autofluorescence. All slides were examined with a fluorescent microscope at x 400 magnification).

The zoites used for the IFAT had autofluorescence, which disrupted the reading of the test. But the fluorescence observed with patient's serum was significantly strong and shiny; and could be easily differentiated from autofluorescence of the parasites (Figure 3). Unfortunately, the DNA extraction of the patient sample obtained from the peripheral blood smear was problematic and no DNA was observed when extraction solution was run on 1% agarose gel. However, PCR amplification of 18S rRNA gene fragment of the parasite using specific primers was performed. No PCR product was detected for the sample. She had been discharged from the hospital without any prescription and her symptoms gradually declined.

However, definitive confirmation of this infection should be obtained by observing the parasite in muscle biopsy and molecular analysis method which we were not able to do because the case did not agree to provide muscle biopsies and the DNA was not extracted from her peripheral blood smear. On the other hand, the clinical manifestations of the case were comparable with patients reported by Abubakar et al. who demonstrated that fever (having relapse nature in 57% of patients) and myalgia were the most frequent symptoms and increase of eosinophil counts (1.0–2.6 × 10^9^ cells/ L) in those patients (3). Furthermore, the parasite in her peripheral blood smear was morphologically similar to merozoites of *Sarcocystis.* In addition, the merozoites of *Sarcocystis* were agglutinated by the case serum. Although, direct agglutination test lacked specificity and the case serum might have antibodies against other coccidian parasite reacting to *Sarcocystis* species (12) but no history of malaria and *Toxoplasma* infections in her might be indicating true positive result of the test.

In conclusion, this work suggested that *Sarcocystis*-like infection should be considered as one of the possible causes of some idiopathic febrile diseases with muscle complications. Consideration of muscular sarcocystosis should be mentioned in differential diagnosis of eosinophilia in areas where the high prevalence of *Sarcocystis* infection is observed in animals. Also, medical laboratory technicians should be alert to this parasite and keep such samples for molecular analysis.
